# Protective Contribution of Rosmarinic Acid in Rosemary Extract Against Copper-Induced Oxidative Stress

**DOI:** 10.3390/antiox13111419

**Published:** 2024-11-19

**Authors:** Arian Kola, Ginevra Vigni, Stefania Lamponi, Daniela Valensin

**Affiliations:** Department of Biotechnology, Chemistry and Pharmacy, University of Siena, Via Aldo Moro 2, 53100 Siena, Italy; arian.kola@unisi.it (A.K.); ginevra.vigni2@unisi.it (G.V.); stefania.lamponi@unisi.it (S.L.)

**Keywords:** rosmarinic acid, natural bioactive compound, copper, neurodegeneration, Alzheimer disease, oxidative stress, reactive oxygen species (ROS), *Rosmarinus offcinalis*, neuroprotection

## Abstract

Rosemary extract (*Rosmarinus officinalis*) is a natural source of bioactive compounds with significant antioxidant properties. Among these, rosmarinic acid is celebrated for its potent antioxidant, anti-inflammatory, antimicrobial, and neuroprotective properties, making it a valuable component in both traditional medicine and modern therapeutic research. Neurodegenerative diseases like Alzheimer’s and Parkinson’s are closely linked to oxidative damage, and research indicates that rosmarinic acid may help protect neurons by mitigating this harmful process. Rosmarinic acid is able to bind cupric ions (Cu^2+^) and interfere with the production of reactive oxygen species (ROS) produced by copper through Fenton-like reactions. This study aims to further evaluate the contribution of rosmarinic acid within rosemary extract by comparing its activity to that of isolated rosmarinic acid. By using a detailed approach that includes chemical characterization, antioxidant capacity assessment, and neuroprotective activity testing, we have determined whether the combined components in rosemary extract enhance or differ from the effects of rosmarinic acid alone. This comparison is crucial for understanding whether the full extract offers added benefits beyond those of isolated rosmarinic acid in combating oxidative stress and Aβ-induced toxicity.

## 1. Introduction

Oxidative stress, caused by an imbalance between reactive oxygen species (ROS) and the body’s antioxidant defenses, plays a crucial role in the aging process and the development of neurodegenerative diseases. Over time, the accumulation of oxidative damage to cellular components like lipids, proteins, and DNA leads to impaired cellular function and increased vulnerability to neurodegeneration. In conditions such as Alzheimer’s, Parkinson’s, and amyotrophic lateral sclerosis (ALS), oxidative stress accelerates neuronal damage, contributing to the progression of these pathologies [1,2,3,4,5,6]. The etiology of these disorders is multifactorial, involving a complex interplay between oxidative stress, mitochondrial dysfunction, metal ion dyshomeostasis, neuroinflammation, and protein misfolding [3,7,8,9,10,11]. Despite significant research efforts, there are currently no definitive cures, highly effective treatments, or preventive therapies for these conditions.

Transition metals, particularly redox-active metals such as copper (Cu) and iron (Fe), are central to this pathogenic network [12,13,14,15,16,17,18,19,20]. These metals are vital for numerous cellular functions, including synaptic transmission and antioxidant defense. However, under disease conditions, excessive accumulation or inappropriate localization of metals like iron and copper can catalyze the production of reactive oxygen species (ROS) via Fenton and Haber–Weiss reactions. This metal-catalyzed oxidative stress is strongly implicated in both the initiation and progression of neurodegenerative diseases, creating a vicious cycle of cellular injury. For instance, copper and iron binding to Amyloid-β (Aβ) in Alzheimer’s Disease (AD) and α-synuclein in Parkinson’s Disease (PD) leads to the production of ROS, which exacerbates neurotoxicity [5,21,22,23]. In vitro studies demonstrate that the Aβ-Cu complex catalyzes the formation of hydrogen peroxide (H_2_O_2_) and hydroxyl radicals (HO•) in the presence of oxygen and reducing agents [24]. Recent research has also identified superoxide as an intermediate species in this metal-catalyzed ROS production, revealing new insights into the molecular pathways contributing to oxidative damage [25].

Elevated radical production is also evident in PD, where copper has been shown to contribute to the generation of ROS by enhancing dopamine oxidation, a process facilitated by the protein α-synuclein [26,27,28,29]. Copper’s role in promoting α-synuclein aggregation further enhances ROS production, leading to protein damage and accelerating neuronal death [29,30].

Given the detrimental effects of metal-catalyzed ROS production, targeting these processes through exogenous antioxidants is a promising prevention and therapeutic strategy. Polyphenols, a diverse group of plant-derived metabolites, have garnered attention for their antioxidant properties and neuroprotective effects [31,32,33,34,35,36,37,38,39]. These compounds, widely present in fruits, vegetables, and whole grains, can scavenge free radicals due to their highly conjugated systems and hydroxylation patterns. Moreover, polyphenols like curcumin, caffeic acid, ferulic acid, and rosmarinic acid have shown strong abilities to bind metal ions, such as copper and iron, thereby inhibiting their role in catalyzing ROS formation [13,40,41,42]. Additionally, polyphenols have been shown to interact with amyloidogenic proteins, such as Aβ and α-synuclein, and inhibit their aggregation, further underscoring their potential therapeutic and preventive role in neurodegeneration [43,44,45].

**Figure 1:**
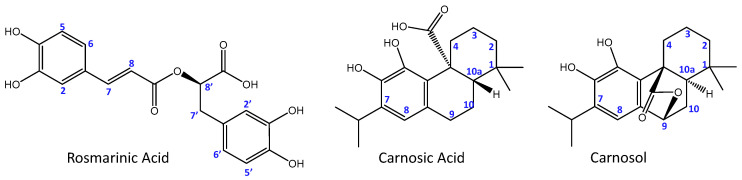
Chemical structure of the main antioxidant compounds in rosemary extracts.

Natural extracts, particularly those rich in polyphenols, have gained increasing attention in recent years due to their potent antioxidant and neuroprotective properties. Historically, such extracts were the primary form of medicine used to treat various diseases before the advent of modern pharmaceuticals, highlighting their long-standing therapeutic value and the importance of rediscovering their potential in contemporary medicine [46]. Among these, rosemary extract (from *Rosmarinus officinalis*) stands out for its high polyphenol content and its effectiveness as a free radical scavenger [47,48,49,50]. The health-promoting properties of rosemary have been attributed to its wide range of bioactive compounds, including rosmarinic acid, carnosic acid, and carnosol, all of which contribute to its strong antioxidant activity [51,52,53,54]. Rosmarinic acid (Figure 1) is a phenolic acid able to inhibit the formation of amyloid aggregates in vitro and to form stable ternary adducts with Amyloid-β and Cu^2+^ ions [55]. Carnosic acid and its derivative carnosol (Figure 1) are both phenolic diterpenes able to neutralize cell membrane damage due to lipid peroxidation and activate the transcription factor regulating the expression of antioxidant proteins.

To gain a deeper understanding of the chemical compounds in rosemary that act as radical scavengers and neuroprotectors against Aβ-induced toxicity, this study compared the antioxidant and neuroprotective activities of *Rosmarinus officinalis* extract (ROE) and rosmarinic acid (RA). Chemical characterization of the extract was performed using standard protocols for the analysis of natural extracts, with quantitative nuclear magnetic resonance (qNMR) spectroscopy enabling the quantification of RA in the extract.

Antioxidant activity tests evaluated the scavenging effects of both RA and the full extract on hydrogen peroxide and ROS species (superoxide anion, hydrogen peroxide, and hydroxyl radical) generated by the ascorbate/copper system. Additionally, cellular assays were conducted to assess the cytotoxicity of the systems analyzed and to evaluate their protective effects against Aβ-induced toxicity. This enabled a comparison between the activities of the full extract and RA at various concentrations to clarify the distinct contribution of RA to the overall antioxidant and neuroprotective effects.

## 2. Materials and Methods

### 2.1. Reagents

CuSO_4_ solution, ascorbic acid (≥99%), rosmarinic acid (≥98.0% HPLC), caffeic acid (≥98.0% HPLC), p-coumaric acid (≥98.0% HPLC)DMSO-d_6_, EtOD, MeOD, CDCl_3_, deuterated TMSP, and phosphate buffer were all supplied by Sigma-Aldrich (Schnelldorf, Germany). Amyloid-β (1–16) peptide (Aβ16) was supplied by DBA Italia (Milano, Italy) and Amyloid-β (1–42) peptide (Aβ42) by Genscript (Rijswijk, The Netherlands). Dulbecco’s Modified Eagle’s Medium, trypsin solution, and all the solvents used for cell culture were purchased from Merck (Darmstadt, Germany). NIH3T3 murine fibroblasts and SH-SY5Y cells were from the American Type Culture Collection (Manassas, VA, USA).

#### Preparation of *Rosmarinus officinalis* Extracts

Rosemary leaves were collected and dried at room temperature for two days. Subsequently, they were coarsely chopped to increase the surface area exposed to the water-ethanol mixture (60:40). The ratio of rosemary to the solvent mixture used was 1 part plant material to 10 part hydroalcoholic solution. Maceration was conducted in the absence of light and at room temperature. The mixture was stirred to ensure complete saturation of the plant material, then covered and left to sit for 7 days, with occasional stirring to enhance the extraction process. After the maceration period, the mixture was filtered using a Büchner funnel to separate the liquid extract from the solid plant material. The filtered liquid extract was collected in a clean glass container, and the solvent was evaporated by gently eating the extract at T = 40 °C or under nitrogen flow at T = 25 °C until a fine powder was obtained. According to the evaporation procedures, two different extracts were obtained, which will be referred to for convenience as hot and cold rosemary extracts, respectively. Both extracts were stored in an amber glass bottle at T = −20 °C. The amount of dry extract obtained from each gram of dried rosemary subjected to extraction was 129 mg for the hot extract and 125 mg for the cold extract, corresponding to percentage yields of 12.9% and 12.5%, respectively.

### 2.2. Chemical Analysis

#### 2.2.1. Determination of Total Phenolic and Flavonoid Content

The total polyphenols and flavonoid content of *Rosmarinus officinalis* hydroethanolic extract was examined using spectrophotometric methods reported by Lamponi et al. [56]. In detail, total polyphenols were determined by the colorimetric method of Folin–Ciocalteu: 0.01 mL of each extract were added to 2.99 mL of distilled water and 0.5 mL of Folin–Ciocalteu reagent 1:10 *v*/*v* in distilled water. After 30 s of shaking, 1.0 mL of Na_2_CO_3_ 15% m/m in distilled water was added. After incubation at room temperature for 120 min, absorbance at 700 nm was read using a Varian Cary 1E. The polyphenol quantification was calculated by means of interpolation of a calibration curve constructed using gallic acid. The total flavonoid content of extracts was determined by reading absorbance at 353 nm of 100-fold diluted extract according to Sosa et al. [57] and constructing a calibration curve using hyperoside as standard.

#### 2.2.2. Determination of Total Triterpenes

A total of 0.01 mL aliquot of the sample solution was added to 0.19 mL of glacial acetic acid, followed by the addition of 0.3 mL of a 5% *w*/*v* vanillin solution in glacial acetic acid. After mixing for 30 s, 1 mL of perchloric acid was introduced into the mixture. The mixture was heated to 60 °C for 45 min, and after cooling, the volume was brought to 5 mL with glacial acetic acid [58]. The absorbance was read at 548 nm, and the quantification of the total triterpenes in the extract was calculated according to the calibration curve constructed using β-sitosterol.

#### 2.2.3. Qualitative and Quantitative Analysis of Rosemary Extract by NMR Spectroscopy

NMR spectra were performed with a Bruker Avance III Spectrometer at 14.1 T and using a 5 mm BBI probe. All the experiments were collected and carried out at the controlled temperature of 298 K ± 0.2 K. Chemical shifts were referenced to external 2-(Trimethylsilyl)-propionic-2,2,3,3-d_4_ acid sodium salt (TMSP-d_4_). One-dimensional and two-dimensional spectra were recorded by using standard pulse sequences and analyzed by using the TopSpin 4.1.4 software. The residual water signal was suppressed by an excitation sculpting pulse program, applying a selective 2 ms long square pulse on water [59]. The NMR samples of rosemary extract were prepared by dissolving the dried extract in different solvents at the following concentrations: 0.44 mg/mL (H_2_O:D_2_O 9:1), 0.626 mg/mL (H_2_O:D_2_O:DMSO-d_6_ (8:1:1), 0.83 mg/mL (H_2_O:EtOD:D_2_O, 7.5:1.5:1), 2.5 mg/mL (MeOD:D2O 9:1), 2.5 mg/mL (DMSO-d_6_), and 2.5 mg/mL (CDCl_3_). The quantification of RA in rosemary extracts was performed by using qNMR [60,61,62]. The NMR spectra of RA were recorded at five different concentrations ranging from 50 to 150 µM. For each concentration, 1D ^1^H NMR experiments were recorded by using the following parameters: SW = 12 ppm, T = 32 K, d1 = 5 s, NS = 512. The obtained spectra were then compared with the ones of rosemary extracts recorded at the same experimental conditions.

### 2.3. Antioxidant Activity

#### 2.3.1. Hydrogen Peroxide Scavenging Assay

The capacity of the tested compounds to scavenge H_2_O_2_ was determined by monitoring H_2_O_2_ absorbance by UV–Vis spectroscopy, as previously reported for different plant extracts [56,63]. Varying concentrations of each compound (rosemary extract, rosmarinic acid, caffeic acid, and p-coumaric acid) were added to a 2 mM H_2_O_2_ solution prepared in a 50 mM phosphate buffer at pH 7.4. All the mixtures were vortexed and analyzed by UV–Vis spectroscopy. The absorbance of H_2_O_2_ was measured at 230 nm following 10 min of incubation. A blank consisting of phosphate buffer and 60% ethanol without H_2_O_2_ was used for comparison. The percentage of scavenged hydrogen peroxide was calculated using the formula:

% scavenged H_2_O_2_ = [(A_i_ − A_t_)/A_i_] × 100 (1)

where A_i_ represents the absorbance of the control and A_t_ denotes the absorbance of the test samples.

#### 2.3.2. Copper-Catalyzed Reactive Oxygen Species

Metal ions, including Cu^2+^ and Fe^3+^, can enhance the oxidation of ascorbate in the presence of oxygen, resulting in the generation of ROS through Fenton-like reactions [64,65]. Typically, the consumption of ascorbate is tracked by measuring its absorbance at 265 nm over time, yielding a characteristic kinetic curve where the slope correlates directly with the reaction rate. A quicker rate of ascorbate oxidation indicates a greater production of ROS.

All stock aqueous solutions of ascorbic acid, rosemary extracts, rosmarinic acid, and Aβ16 and CuSO_4_ were freshly prepared and subsequently diluted with phosphate buffer and distilled water to achieve the desired concentration in the cuvette with a total volume of 0.5 mL. The absorption spectra and kinetic curves (over 45 min, or 2700 s) were recorded using an Agilent Cary UV–Vis spectrophotometer. The absorbance at 265 nm was then plotted as a line graph by using Origin Pro 2018 software.

### 2.4. Cellular Studies

#### 2.4.1. NIH3T3 Cytotoxicity

The in vitro cytotoxicity of the compounds was evaluated by the direct contact test towards NIH3T3 cells. NIH3T3 cells were propagated in DMEM supplemented with 10% fetal calf serum, 1% L-glutamine–penicillin–streptomycin solution, and 1% MEM non-essential amino acid solution and incubated at 37 °C in a humidified atmosphere containing 5% CO_2_. Once at the confluence, the cells were washed with PBS 0.1M, separated with a trypsin-EDTA solution, and centrifuged at 1.000 r.p.m. for 5 min. The pellet was resuspended in complete medium (dilution 1:15). Cells (1.5 × 10^4^) suspended in 1 mL of complete medium were seeded in each well of a 24-well round multidish and incubated at 37 °C in an atmosphere of 5% CO_2_. Once reached 50% of confluence (i.e., after 24 h of culture), the culture medium was discharged, and the test compounds, properly diluted in the completed medium, were added to each well. All samples were set up in six replicates. A complete medium was used as a negative control. After 24 h of incubation, cell viability was evaluated by a neutral red uptake (NRU) assay [66].

#### 2.4.2. Neuroprotective Activity of *Rosmarinus officinalis* Extract

SH-SY5Y cells were differentiated in neurons following the procedure previously reported [67,68] in Petri dishes (35 mm ∅) with a density of about 3600 cells/mm^2,^ according to Biffi et al. [69]. Then, at each Petri dish, 2 mL of appropriate culture medium was added containing each test sample and incubated for 24 h at 37 °C in the atmosphere with 5% CO_2_. Each sample was tested in triplicate. At the end of the incubation, the viability of the differentiated SH-SY5Y cells was evaluated using an NRU assay [66].

#### 2.4.3. Statistical Analysis of In Vitro Cell Tests

Multiple comparisons were conducted using one-way ANOVA, with individual differences assessed using Fisher’s test following the identification of significant intergroup differences by ANOVA. Differences were deemed significant at *p* < 0.05.

## 3. Results

### 3.1. Chemical Characterization of Rosmarinus officinalis Extracts

The phenolic, flavonoid, and triterpene contents of two *Rosmarinus officinalis* extracts (ROEs) were analyzed using standard protocols, as described in the Section 2. Two extraction techniques were employed and compared to evaluate the impact of different evaporation methods on extraction efficiency. The results, shown in Table 1, detail the total phenolic content in gallic acid equivalents (GAE), flavonoid content in hyperoside equivalents, and triterpene content in β-sitosterol equivalents. These concentrations are expressed in milligrams per gram of dry extract (mg/g d.e.), allowing for consistent comparisons of the bioactive compounds crucial for evaluating the extracts’ potential antioxidant and therapeutic properties.

Initially, it was expected that evaporation under nitrogen would preserve more polyphenols and volatile compounds by reducing oxidation. However, the data reveal that both methods yielded similar concentrations of phenolic, flavonoid, and triterpene compounds, suggesting that the evaporation method does not significantly influence the concentration of these bioactive components. This indicates that both methods are equally effective in extracting the key bioactive constituents, with the main difference being the longer processing time required for nitrogen evaporation. Furthermore, these findings are consistent with previous studies conducted on similar hydroalcoholic extracts of Tuscan *Rosmarinus officinalis* [56].

**Figure 2:**
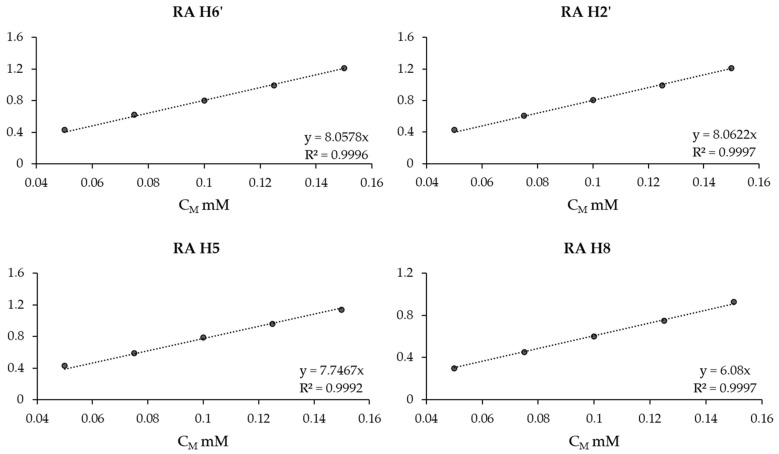
Example of calibration curves obtained for H2, H6, H2’ and H5’ signals of RA.

To gain a deeper understanding of the primary bioactive components in ROEs, an NMR analysis was conducted. This analysis provided detailed insights into the molecular structure and composition of the extracts. The NMR study was performed using a variety of solvents, including water, ethanol (EtOD), methanol (MeOD), dimethyl sulfoxide (DMSO-d_6_), and chloroform (CDCl_3_), to ensure that both hydrophilic and hydrophobic components were detectable in the NMR spectra. As expected, the appearance of the signals was highly dependent on the solvent used. DMSO-d_6_, in particular, served as an intermediate solvent, effectively solubilizing compounds with both polar and non-polar characteristics (Figure S1). Given that rosemary extract is commonly formulated in hydroalcoholic solutions, the chemical characterization was further conducted by analyzing NMR spectra recorded only in aqueous or alcoholic solutions. Among the main constituents, rosmarinic acid is well known as the most abundant polyphenol in ROEs [56,70]. The corresponding aromatic protons are easily observable in all the recorded NMR spectra of ROEs, except for those recorded in CDCl_3_. A detailed and clear identification of the rosmarinic acid resonances was obtained by comparing the spectra of the pure compound to those of the extracts recorded at the same experimental conditions (Figure S2). Therefore, the content of RA in ROEs was determined using quantitative nuclear magnetic resonance (qNMR) by recording 1D ^1^H NMR spectra of RA at five different concentrations (50 µM, 75 µM, 100 µM, 125 µM, and 150 µM). These data were then used to construct calibration curves (Figure 2). The area under the signals corresponding to all RA protons was measured and plotted on a graph, which was then used to determine the concentration of RA in the ROE samples by comparing the measured signal area of the ROE samples (Table 2). The average values obtained for hot and cold extracts are 76 and 78 µM, respectively. These values are very similar, and importantly, when considering the standard deviations, which are approximately ±1, there is no significant difference between the two measurements. This reinforces the conclusion that both extraction methods yield comparable concentrations of the target compounds, indicating that the choice of extraction technique does not substantially affect the overall composition of the extracts.

In addition to RA, the NMR analysis of the hydroalcoholic and aqueous solutions of the ROEs revealed the presence of other compounds, including phenolic diterpenoids such as carnosic acid, carnosol, and their derivatives, exhibiting specific scalar correlations between the methine proton (CH) at 3.17 ppm and the terminal methyl protons (CH_3_) of the isopropyl group at position 7, respectively. The inability to uniquely identify the signals of these molecules prevented monitoring the concentration of this important class of compounds known for their pronounced antioxidant activity and high neuroprotective effect [71]. However, the NMR analysis revealed that the content of these diterpenoids is lower compared to RA, which is in agreement with previous studies [56,70]. Finally, the ability of ROEs to interact with Cu^2+^ was investigated by examining the copper-induced line broadening of the ^1^H NMR signals of the extract upon the addition of increasing amounts of cupric ions. Paramagnetic metals such as copper(II) are known to accelerate the relaxation rates of nuclei near the metal center, leading to significant line broadening of the NMR resonances. Therefore, the NMR signals that exhibit broadening can be attributed to molecules capable of interacting with the cupric ion, as is evident for the signals of rosmarinic acid and the signals at 3.17, belonging to compounds derived from carnosic acid (Figure S3). Additionally, the effect of the cupric ion was observed on the signals at 1.89 ppm, which can be attributed to triterpene glycosides present in the extract, such as ursolic acid and oleanolic acid. Among the four compounds identified and most influenced by the paramagnetic ion, rosmarinic and ursolic acid are indeed known for their ability to bind copper(II) ions [44,55,72].

### 3.2. Antioxidant Activity of Rosmarinus officinalis Extract

All measurements related to the antioxidant activity of ROE were conducted on the hot extract for convenience, considering the shorter solvent evaporation times. The percentage of radical-scavenged hydrogen peroxide as a function of increasing concentrations of ROE was calculated and reported in Table 3. As expected, the scavenging activity increased with the amount of extract. Table 3 also correlates the antioxidant activity with the total content of the main antioxidant compounds found in ROE (phenols, flavonoids, and terpenoids), with polyphenols being the most abundant in all cases. Moreover, to better evaluate the contribution of polyphenolic acids to the antioxidant activity of ROE, the radical scavenging abilities of three individual polyphenolic acids commonly found in *Rosmarinus officinalis* were also assessed, as shown in Table 4.

The data in Table 4 clearly indicate that RA is the most effective in scavenging hydrogen peroxide, showing an activity strongly dependent on its concentration up to 50 μg/mL. On the other hand, caffeic acid and p-coumaric acid exhibit a lower percentage of H_2_O_2_ scavenging, which is, at the same time, less influenced by concentration. Moreover, RA exhibits a behavior that strongly resembles the one reported in Table 3 for ROE (total phenolic content).

**Figure 3:**
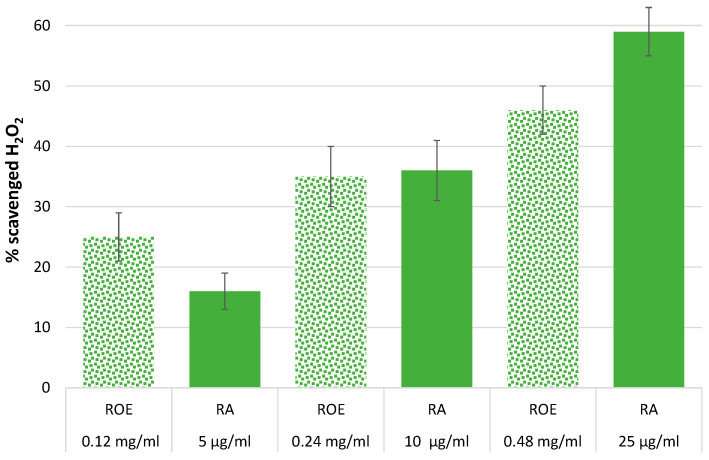
Comparison of the percentage of scavenged hydrogen peroxide between ROE and RA at different concentrations. The concentration of ROE was selected to contain the same amount of RA as used for the comparison. The data reported in Figure are those shown in Table 2 and Table 3.

To better correlate the antioxidant activity of RA and ROE, we extrapolated the amount of rosmarinic acid present in the ROE extract using the values obtained from NMR spectroscopy. Specifically, our data indicate that 1 mg of dried extract contains approximately 40–50 μg of rosmarinic acid, allowing us to correlate the following ROE concentrations: 0.12 mg/mL, 0.24 mg/mL, and 0.48 mg/mL with specific RA amounts, resulting in the following values: 5–6 μg, 9–12 μg, and 19–24 μg. Finally, the percentages of H_2_O_2_ scavenging by ROE were compared with those of RA at concentrations closest to the measured values, as shown in Figure 3.

The data indicate a variable antioxidant activity of ROE across different concentration levels, although comparisons may be affected by experimental variability in the concentration range of RA. Unfortunately, the data do not allow for a clear understanding of RA’s contribution to the overall antioxidant activity of ROE, limiting our ability to assess possible cooperative or competitive interactions between ROE components. Further analysis is needed to clarify these interactions, which will be essential for optimizing rosemary extract in various applications and ensuring its beneficial properties are effectively utilized without unintended effects.

**Figure 4:**
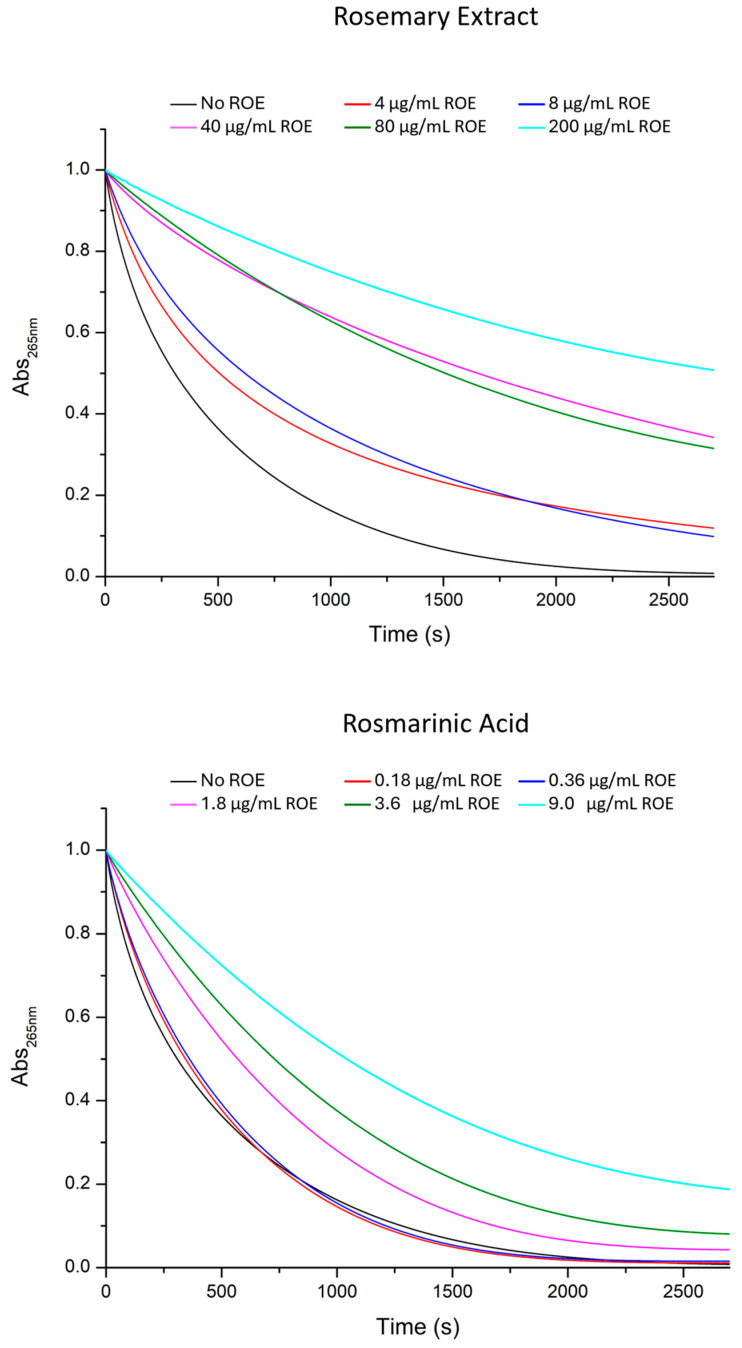
UV–Vis kinetic curves of the systems composed of ascorbate 50 μM, Cu^2+^ 0.5 μM, phosphate buffer 1 mM, and ROE and RA at different concentrations. The black curve represents the ascorbate/Cu^2+^ system alone, while the colored lines correspond to the addition of increasing concentrations of ROE (4 µg/mL—red; 8 µg/mL—blue; 40 µg/mL—magenta, 80 µg/mL—olive and 200 µg/mL—cyan) and RA (0.18 µg/mL—red; 0.36 µg/mL—blue; 1.8 µg/mL—magenta; 3.6 µg/mL—olive and 9 µg/mL—cyan). The concentration of ROE was selected to contain the same amount of RA as used for the comparison.

The antioxidant activity of ROE and its corresponding RA, at the same concentration present in the extract, was further investigated through their ability to interfere with ROS generated by the ascorbic acid and copper(II) system. The consumption of ascorbic acid in these conditions was monitored by measuring its UV–Vis absorption at 265 nm over a period of 45 min. A range of concentrations for both RA and ROE were tested, ensuring that the amount of RA in the ROE matched that used in the RA experiments. Specifically, the concentrations used for RA were 0.18 µg/mL, 0.36 µg/mL, 1.8 µg/mL, 3.6 µg/mL, and 9 µg/mL, while for the extract 4 µg/mL, 8 µg/mL, 40 µg/mL, 80 µg/mL, and 200 µg/mL (0.2 mg/mL) for the extract. The data, as reported in Figure 4, show that higher concentrations consistently led to a slower consumption of ascorbic acid across all systems tested. Notably, the effects were significantly more pronounced in the presence of the full extract compared to RA at all concentrations. ROE was able to slow down copper-induced consumption of ascorbate even at the lowest concentrations, while RA was completely ineffective. Moreover, the antioxidant activity of the ROE was greater than that of RA even at the concentration range (0.20–0.24 mg/mL), which previously resulted in leveling off the values between RA and ROE (Figure 4). This strongly suggests that the extract has a superior ability to counteract ROS, including superoxide, hydrogen peroxide, and hydroxyl radicals, which are generated by ascorbic acid in the presence of copper ions. The more pronounced effect of the full extract compared to rosmarinic acid alone could be attributed to the presence of other compounds in ROE, with polyphenols as likely candidates, although additional compounds may also contribute to the overall antioxidant capacity. Furthermore, polyphenols in the extract might interact with cupric ions (Cu^2+^), potentially slowing the formation of radical species by interfering with the ascorbic acid-copper redox cycle. This interaction could reduce the catalytic activity of copper ions, thus inhibiting the continuous generation of ROS. This combined mechanism—synergistic scavenging of multiple ROS species and direct interference with copper ion activity—might justify the extract’s ability to protect against oxidative stress more effectively than RA.

**Figure 5:**
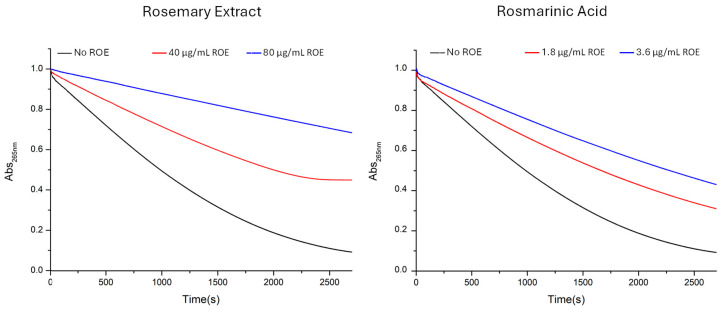
UV–Vis kinetic curves of the systems composed of ascorbate 20 μM, Cu^2+^ 1 μM, Aβ16 10 μM, phosphate buffer 1 mM, and ROE and RA at different concentrations. The black curve represents the ascorbate/Cu^2+^/Aβ16 system alone, while the red and blue lines correspond to the addition of increasing concentrations of ROE (40 µg/mL and 80 µg/mL) and RA (1.8 µg/mL and 3.6 µg/mL).

Identical measurements were conducted using solutions containing amyloid beta peptides, which are well known to generate reactive oxygen species (ROS) in the presence of copper and a reducing agent like ascorbic acid [25,65]. The results obtained are illustrated in Figure 5. As previously noted, the effects of ROE are significantly more pronounced than those exhibited by RA, suggesting that the components of rosemary have the ability to interfere with Aβ-Cu^2+^ complexes in a manner similar to that of RA, which modulates the association of Amyloid β with copper by forming a ternary adduct [55]. The two concentrations of ROE investigated were selected to provide rosmarinic acid concentrations of 1.8 µg/mL (5 µM) and 3.6 µg/mL (10 µM), corresponding to the concentrations shown to be effective in antioxidant activity analyses of rosmarinic acid and the extract in the absence of Aβ (Figure 4).

### 3.3. Cytotoxicity and Neuroprotective Activity of Rosmarinus officinalis Extract

The effects of ROE and RA on NIH3T3 cell viability were evaluated after a 24 h exposure. As shown in Figure S4, RA was not toxic at any of the tested concentrations, while ROE reduced cell viability at concentrations greater than 0.2% *v*/*v*. The same experiments were conducted using Cu^2+^ alone and in combination with ROE. The data reported in Figure S5 indicate that no cytotoxicity was observed in either case. The measurements with copper were performed at the non-toxic concentration of ROE (0.2% *v*/*v*), and it was found that any slight reduction in cell viability caused by the metal ion was restored by the presence of rosemary extract.

**Figure 6:**
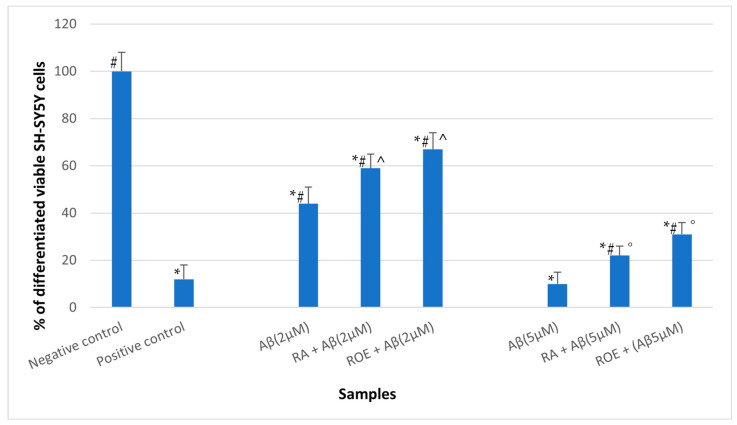
Effect of Aβ 2 µM and 5 µM towards the viability of differentiated SH-SY5Y as a function of RA (0.2% *v*/*v*) and ROE (0.2% *v*/*v*) treatments, as determined by the neutral red uptake. Data are mean ± SD of three replicates for each sample. * Values are statistically different versus negative control (complete medium), *p* < 0.05. # Values are statistically different versus positive control (PVC-org.Sn), *p* < 0.05. ^ Values are statistically different from Aβ 2 µM, *p* < 0.05. ° Values are statistically different from Aβ 5 µM, *p* < 0.05.

The lowest concentrations of ROE and RA were also employed in experiments using differentiated SH-SY5Y cells exposed to Aβ. This cellular model is widely utilized to assess neuroprotective activity against the toxicity of Amyloid β. As expected, a 24 h exposure to Aβ42 significantly reduced cell viability, decreasing it to approximately 10% at a concentration of 2 μM and 40% at 5 μM (Figure 6). In the presence of RA, cell viability increased to 20% and 60%, while ROE resulted in an increase to 30% and 68%. These results indicate that both RA and ROE are effective in mitigating amyloid toxicity in differentiated SH-SY5Y cells, with ROE demonstrating slightly greater effectiveness.

## 4. Discussion

Rosemary extract, derived from the leaves of the aromatic herb *Rosmarinus officinalis*, has garnered significant attention in recent years due to its potent antioxidant properties. Numerous studies have demonstrated that this extract contains a rich array of bioactive compounds, including rosmarinic acid, carnosic acid, and carnosol, which collectively contribute to its impressive antioxidant activity [70,73,74,75,76,77]. One of the primary mechanisms through which ROE exerts its antioxidant effects is by scavenging free radicals, such as superoxide anions and hydroxyl radicals. These reactive species can lead to oxidative stress, which is implicated in various diseases, including neurodegenerative disorders like Alzheimer’s disease [78,79,80,81]. Indeed, several studies have demonstrated the ability of ROE to protect neuronal cells from Aβ toxicity [82,83,84], highlighting its role in preserving cognitive function and enhancing memory and learning capabilities.

Rosemary extract contains various bioactive compounds with differing polarities, and the choice of solvent used in chemical investigations significantly influences the composition of the extract [56,70,85,86]. Specifically, aqueous solutions have been shown to result in a higher concentration of RA, whereas less polar solvents, such as acetonitrile and DMSO, tend to highlight carnosic acid as the predominant component. The different solubility of the rosemary components is clearly evident when comparing the NMR spectra recorded from the same extract dissolved in different solvents (Figure S1), further illustrating how solvent choice influences the extraction and composition of bioactive compounds in rosemary. This variation underscores the importance of solvent selection in the analysis process, as it directly affects the yield and profile of bioactive constituents in rosemary, thereby impacting its potential health benefits and applications. In light of these considerations, this study focused on the chemical analysis and biological activity of a rosemary extract in aqueous solution. Our aim was to elucidate the role of one of its main components, RA, in the antioxidant and neuroprotective activities of the extract.

The rosemary extracts used in our analysis were obtained through two different methods: evaporation under nitrogen flow (cold ROE) and evaporation at 40 °C (hot ROE). The choice of nitrogen flow was driven by the goal of preserving the entire range of polyphenols, particularly the volatile compounds. These bioactive molecules, known for their antioxidant properties, are susceptible to oxidation and thermal degradation when exposed to heat or oxygen. Nitrogen flow provides an oxygen-free environment, preventing oxidative damage and maintaining the integrity of temperature-sensitive polyphenols such as rosmarinic acid. On the other hand, the evaporation at 40 °C was performed to compare the efficiency and preservation capabilities of the two methods. This process, while faster, exposes the extract to a higher temperature and a prolonged oxygen presence, potentially risking the degradation of certain phenolic compounds. Despite these concerns, it was hypothesized that differences in the concentration of phenolic compounds and the antioxidant capacity between the two methods would be evident. Surprisingly, the results indicated that both methods yielded similar concentrations of phenolic compounds, including flavonoids and triterpenes (Table 1 and Table 2 and Figure 2). While the nitrogen flow method initially seemed superior in protecting volatile and heat-sensitive compounds, the analysis demonstrated that evaporation at 40 °C did not significantly alter the chemical composition or bioactivity of the extract. However, one key difference remained in terms of the processing time. The evaporation under nitrogen flow required significantly longer times for solvent removal compared to the 40 °C method, which was more efficient time-wise. For these reasons, we decided to proceed with all analyses of antioxidant and neuroprotective activities using the hot ROE only.

The investigations on the cytoprotective activities were conducted on both the rosemary extract (ROEs) and rosmarinic acid (RA), which was identified as the major component of the extract, as highlighted by NMR analysis (Figure S2). This finding is consistent with previous chemical analyses using experimental protocols similar to ours [70].

The obtained results provide valuable insights into the antioxidant mechanisms of ROE and its major component, RA. The H_2_O_2_-scavenging activities of ROE and RA demonstrate the concentration-dependent efficacy of both the extract and its polyphenolic constituents. As expected, the scavenging activity of ROE increased with concentration, which aligns with the total content of phenolic compounds (Table 3). RA, as the predominant phenolic acid in the extract, demonstrated a significantly stronger H_2_O_2_-scavenging ability compared to other polyphenolic acids, such as caffeic acid and p-coumaric acid (Table 4). This result is consistent with previous studies and confirms RA’s crucial contribution to the overall antioxidant effect of ROE [87]. Interestingly, the comparison between RA alone and its presence in the ROE revealed nuanced behavior at different concentration levels (Figure 3). At lower concentrations, ROE exhibited superior antioxidant activity compared to RA alone, suggesting a potential synergistic interaction between the various bioactive components in ROE. This synergy likely amplifies the scavenging effects of the extract beyond what could be achieved by any single compound, including RA. However, at higher concentrations, RA alone outperformed the extract, implying that the interactions among the compounds in ROE may become competitive or inhibitory at elevated levels, thus diminishing the overall antioxidant efficacy of the extract. These findings underscore the importance of concentration when evaluating the antioxidant potential of plant extracts, as synergistic or competitive interactions can alter the expected outcomes.

Further investigations into the ability of RA and ROE to combat ROS generated by the ascorbate–copper(II) system revealed an even more pronounced difference between the two. While RA alone was not so effective in mitigating the consumption of ascorbic acid in this model, ROE exhibited a strong protective effect, even at low concentrations (Figure 4). This suggests that the full extract possesses a broader spectrum of antioxidant capabilities, likely due to the presence of multiple polyphenolic compounds working together.

It is important to note that in the first test, the only reactive species involved was hydrogen peroxide (H_2_O_2_), whereas in the ascorbate–copper(II) system, multiple ROS, including superoxide and hydroxyl radicals, are generated simultaneously. The presence of these diverse ROS likely explains why ROE performed significantly better in this test compared to RA alone. These findings suggest that the various components in ROE not only act as direct scavengers of different ROS species but also potentially interfere with the redox cycling of copper ions, reducing their catalytic activity and inhibiting continuous ROS production. This dual mechanism—scavenging multiple ROS species and interfering with metal-catalyzed oxidative reactions—highlights the superior antioxidant potential of the full extract compared to rosmarinic acid alone.

NMR analysis of Cu^2+^ interaction with ROE revealed that, besides RA, other components like triterpenoids and phenolic diterpenes also have the ability to interact with the paramagnetic ion (Figure S3). Notably, recent studies have shown that the antioxidant activity of ursolic acid, a triterpenoid found in rosemary, is enhanced in the presence of copper [72]. This indicates that these compounds may contribute to the ROE’s capacity to interact with copper ions, thereby reducing copper catalytic activity in ROS generation.

Finally, we evaluated the protective effect of ROE against Aβ peptide-induced cytotoxicity. NIH3T3 cell lines were used to assess the cytotoxicity of the analyzed systems, serving as a preliminary step before conducting experiments with differentiated SH-SY5Y cells. As expected, SH-SY5Y cells treated with Aβ42 exhibited a concentration-dependent decrease in cell viability. However, Aβ cotreatment with RA and ROE significantly reduced cell mortality, with ROE demonstrating slightly higher efficacy than RA (Figure 6). These data are in strong agreement with the findings from assays measuring ROS generated by the Aβ-Cu^2+^ system in the presence of ascorbic acid, where ROE again demonstrated greater effectiveness than RA (Figure 5).

All these findings support the notion that the synergistic effects of the various bioactive components within ROE contribute to its enhanced protective capacity and suggest that the components of ROE work collaboratively to mitigate both cell death and oxidative stress in agreement with previous observations [76,77,88]. Future investigations should focus on identifying specific interactions among the components of ROE and their individual contributions to the overall neuroprotective effects observed in this work. In fact, while this study provides valuable insights into the antioxidant and neuroprotective properties of rosemary extract, there are some limitations to address. Primarily, the specific contributions and mechanisms of individual components within ROE remain unclear, as does their precise interaction with Aβ and Cu^2+^. Future research should aim to isolate and characterize these individual bioactive compounds to determine their roles in modulating oxidative stress and neurotoxicity. Additionally, investigating the effects of ROE in vivo models of neurodegeneration would further clarify its therapeutic potential and applicability.

## 5. Conclusions

This study provides a comprehensive evaluation of the antioxidant and neuroprotective properties of rosemary extract and its principal component, rosmarinic acid. Our findings highlight the superior efficacy of ROE in mitigating oxidative damage, primarily through its ability to scavenge a broad range of ROS generated in the ascorbic acid and Cu^2+^ systems. Compared to RA alone, ROE exhibited a more pronounced inhibitory effect on ROS generation, even at lower concentrations. This enhanced activity is likely the result of synergistic interactions among the various polyphenolic and terpenoid compounds present in the extract, which not only scavenge ROS directly but also interact with copper ions, thereby modulating the redox cycling that drives continuous ROS production.

Additionally, ROE demonstrated significant neuroprotective activity in differentiated SH-SY5Y cells exposed to Aβ42, a model for neurodegenerative diseases such as Alzheimer’s. Both ROE and RA reduced Aβ-induced cell death in a concentration-dependent manner. The ability of ROE to protect against Aβ-Cu^2+^-induced ROS formation further suggests that the complex mixture of bioactive compounds within the extract works through multiple mechanisms to mitigate both oxidative stress and metal ion toxicity, offering a broader scope of protection than RA alone.

Rosemary extract can exert a dual beneficial role by both neutralizing ROS and interfering with copper binding to Aβ. It is well established that Aβ-Cu(II) complexes promote Aβ misfolding into toxic species and enhance oxidative stress. Previous studies have shown that rosmarinic acid, a main constituent of rosemary extract, can interfere with Cu(II) binding to Aβ, forming a ternary adduct that may protect against Aβ misfolding [55]. Furthermore, our findings strongly support the ability of rosmarinic acid to scavenge ROS produced by Aβ-Cu(II) complexes in the presence of reducing agents, such as ascorbic acid.

Overall, this study underscores the therapeutic potential of rosemary extract as a powerful antioxidant and neuroprotective agent. The combined action of its constituents offers a multifactorial approach to combating oxidative damage and neurotoxicity, which could have significant implications for research aimed at preventing or mitigating the progression of neurodegenerative diseases. Further studies should focus on elucidating the specific contributions of individual components within ROE and exploring their interactions with key molecular targets involved in oxidative stress and neurodegeneration.

## Figures and Tables

**Table 1:** Extraction yield, total phenolic, flavonoids, and triterpenoids content. Results are expressed as mean value ± standard deviation (SD) from three different preparations.

	HOT ROE	COLD ROE
Total Phenolic Content (mg GAE/g d.e. ± SD)	87.7 ± 1.8	82.2 ± 2.1
Total Flavonoids Content (mg Hyperoside/g d.e. ± SD)	5.4 ± 1.1	4.9 ± 0.9
Total Triterpenoids Content (mg b-sitosterol/g d.e. ± SD)	67.3 ± 4.7	64.1 ± 5.9

**Table 2:** Quantification of RA concentration in hot and cold ROEs using NMR signal area and calibration curves.

H Type	Area in ROE		Slope of the Calibration Curves of RA	[RA] in ROEs (µM) ^1^	
	HOT	COLD		HOT	COLD
H6	0.53	0.51	7.36	72	60
H2	0.59	0.54	7.76	76	70
H6’	0.65	0.61	8.06	80	76
H5	0.70	0.73	7.75	90	94
H2’	0.50	0.49	8.06	62	61
H5’	0.62	0.62	8.16	76	76
H8	0.43	0.51	6.08	71	84
H7’	0.66	0.57	7.64	86	75
H7’	0.74	0.75	7.83	95	96
H7	0.44	0.46	6.31	68	73
				Average value	Average value
				76 ± 1.2	78 ± 1.0

^1^ The concentration of RA was quantified by correlating the area of the NMR signals of RA in the ROEs with the calibration curves shown in Figure 2. The final concentration is reported as the mean value, along with the standard deviation.

**Table 3:** Total phenolic content and percentage of scavenged hydrogen peroxide as a function of increasing concentrations of ROE.

Concentration of d.e. (mg/mL)	% Scavenged H_2_O_2_ ± SD	Total Phenolic Content (mg/mL)	Total Flavonoid Content (mg/mL)	Total Triterpenoids (mg/mL)
0.024	12 ± 2	2.1 × 10^−3^	1.3 × 10^−4^	1.6 × 10^−3^
0.12	25 ± 4	1.1 × 10^−2^	6.5 × 10^−4^	8.1 × 10^−3^
0.24	35 ± 5	2.1 × 10^−2^	1.3 × 10^−3^	1.6 × 10^−2^
0.48	46 ± 4	4.2 × 10^−2^	2.6 × 10^−3^	3.2 × 10^−2^
0.72	57 ± 4	6.3 × 10^−2^	3.9 × 10^−3^	4.8 × 10^−2^
0.96	63 ± 5	8.4 × 10^−2^	5.2 × 10^−3^	6.5 × 10^−2^

**Table 4:** Percentage of hydrogen peroxide of rosmarinic acid, caffeic acid, and p-coumaric acid at different concentrations.

Polyphenolic Acid	Concentration		% Scavenged H_2_O_2_ ± SD
Rosmarinic Acid	5 μg/mL 10 μg/mL 25 μg/mL 50 μg/mL 100 μg/mL	(14 µM) (28 µM) (70 µM) (140 µM) (280 µM)	16 ± 3
			36 ± 5
			59 ± 4
			77 ± 6
			83 ± 5
Caffeic Acid	100 μg/mL 200 μg/mL 300 μg/mL 400 μg/mL 500 μg/mL	(550 µM) (1.1 mM) (1.6 mM) (2.2 mM) (2.8 mM)	17 ± 4
			23 ± 3
			26 ± 5
			32 ± 4
			29 ± 3
p-Cumaric Acid	10 μg/mL 20 μg/mL 30 μg/mL 40 μg/mL 50 μg/mL	(60 µM) (120 µM) (180 µM) (240 µM) (300 µM)	31 ± 3
			36 ± 4
			39 ± 3
			41 ± 3
			40 ± 2

## Data Availability

Data are contained within the article and Supplementary Materials.

## Supplementary Materials

The following supporting information can be downloaded at: https://www.mdpi.com/article/10.3390/antiox13111419/s1, Figure S1: Aromatic (upper) and aliphatic regions of ^1^H NMR spectra of hot ROE in different solvents. T = 298 K. The prominent signals marked with an asterisk at 7.26, 3.15, 2.70, and 2.50 ppm correspond to the deuterated solvents. Figure S2: Aromatic regions of ^1^H NMR spectra of rosmarinic acid (black trace), cold (magenta), and hot (blue) rosemary extracts in water. T = 298 K. The signals of rosmarinic acid are marked with an asterisk. Figure S3: Selected regions of ^1^H NMR spectra of rosemary extract (black trace) in the presence of an increasing amount of Cu^2+^ (colored traces). Figure S4: Percentage of viable NIH3T3 after 24 h of contact with different concentrations of RA and ROE as determined by the neutral red uptake. Data are the mean SD of three experiments run in six replicates. Figure S5: Percentage of viable NIH3T3 after 24 h of contact with ROE 1 mg/mL 0.2% (*v*/*v*), Cu^2+^ 5 mM 0.2% (*v*/*v*), and ROE+Cu^2+^ as determined by the neutral red uptake. Data are the mean SD of three experiments run in six replicates.
