# Supplementary material for: Protective Contribution of Rosmarinic Acid in Rosemary Extract Against Copper-Induced Oxidative Stress
Source: Antioxidants (Basel). 2024 Nov 19;13(11):1419. doi: 10.3390/antiox13111419 (PMC11590892; doi:10.3390/antiox13111419)
Supplement: Supplementary file 1 [file antioxidants-13-01419-s001.zip › antioxidants-3273016-supplementary.pdf]

Supplementary Materials

Protective Contribution of Rosmarinic Acid in Rosemary Extract Against Copper-Induced Oxidative Stress

Arian Kola, Ginevra Vigni, Stefania Lamponi and Daniela Valensin*

Department of Biotechnology, Chemistry and Pharmacy, University of Siena, Via Aldo Moro 2, 53100 Siena, Italy; arian.kola@unisi.it, ginevra.vigni2@unisi.it, stefania.lamponi@unisi.it, daniela.valensin@unisi.it

*Correspondence: daniela.valensin@unisi.it; Tel.: +39-0577-232428

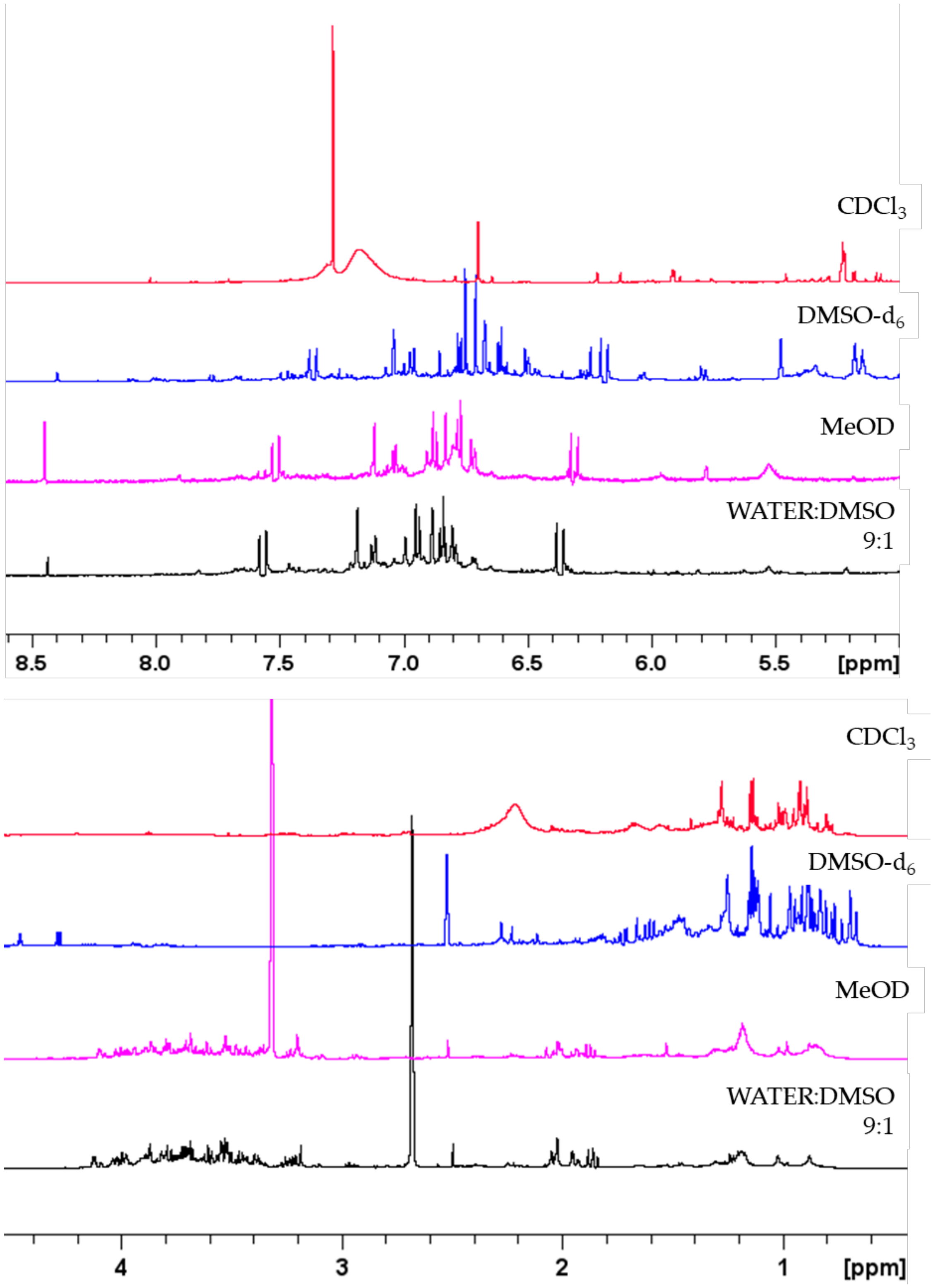

Figure S1. Aromatic (upper) and aliphatic regions of ^{1}H NMR spectra of hot ROE in different solvents. T=298 K. The prominent signals marked with an asterisk at 7.26, 3.15, 2.70, and 2.50 ppm correspond to the deuterated solvents.

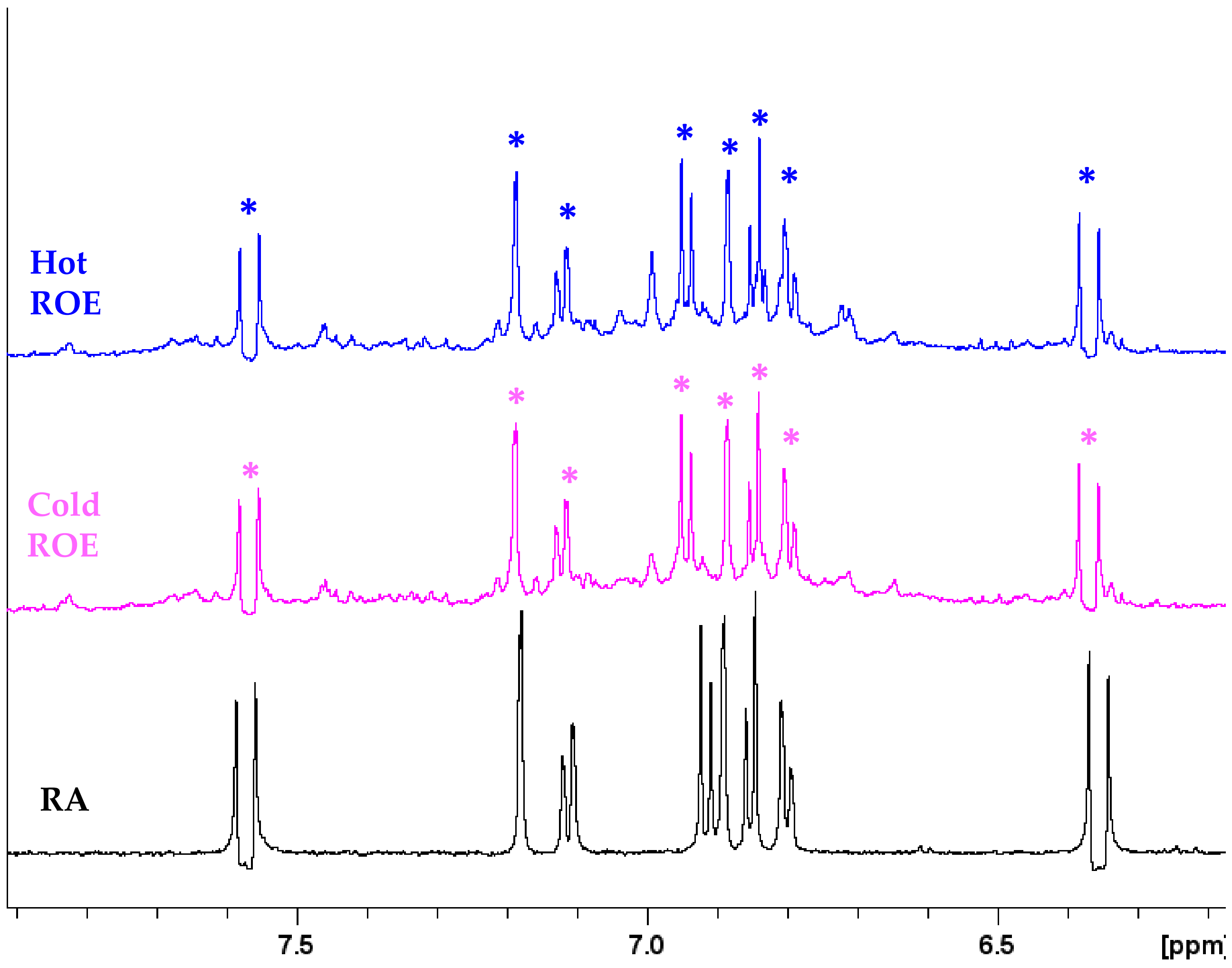

Figure S2. Aromatic regions of ^{1}H NMR spectra of rosmarinic acid (black trace), cold (magenta) and hot (blue) Rosemary extracts in water. T=298 K. The signals of rosmarinic acid are marked with an asterisk.

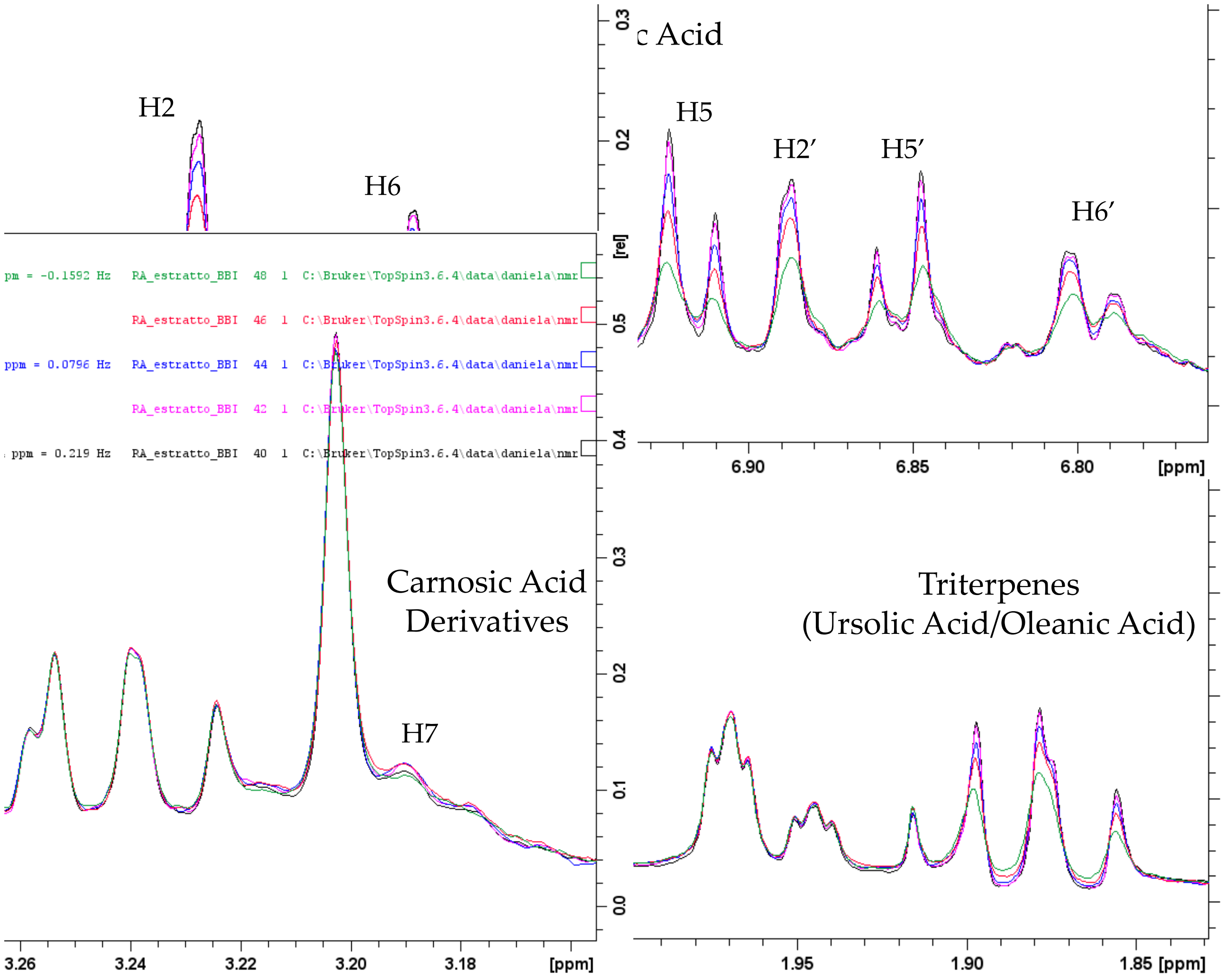

Figure S3. Selected regions of ^{1}H NMR spectra of rosemary extract (black trace) in presence of increasing amount of Cu^{2+} (colored traces).

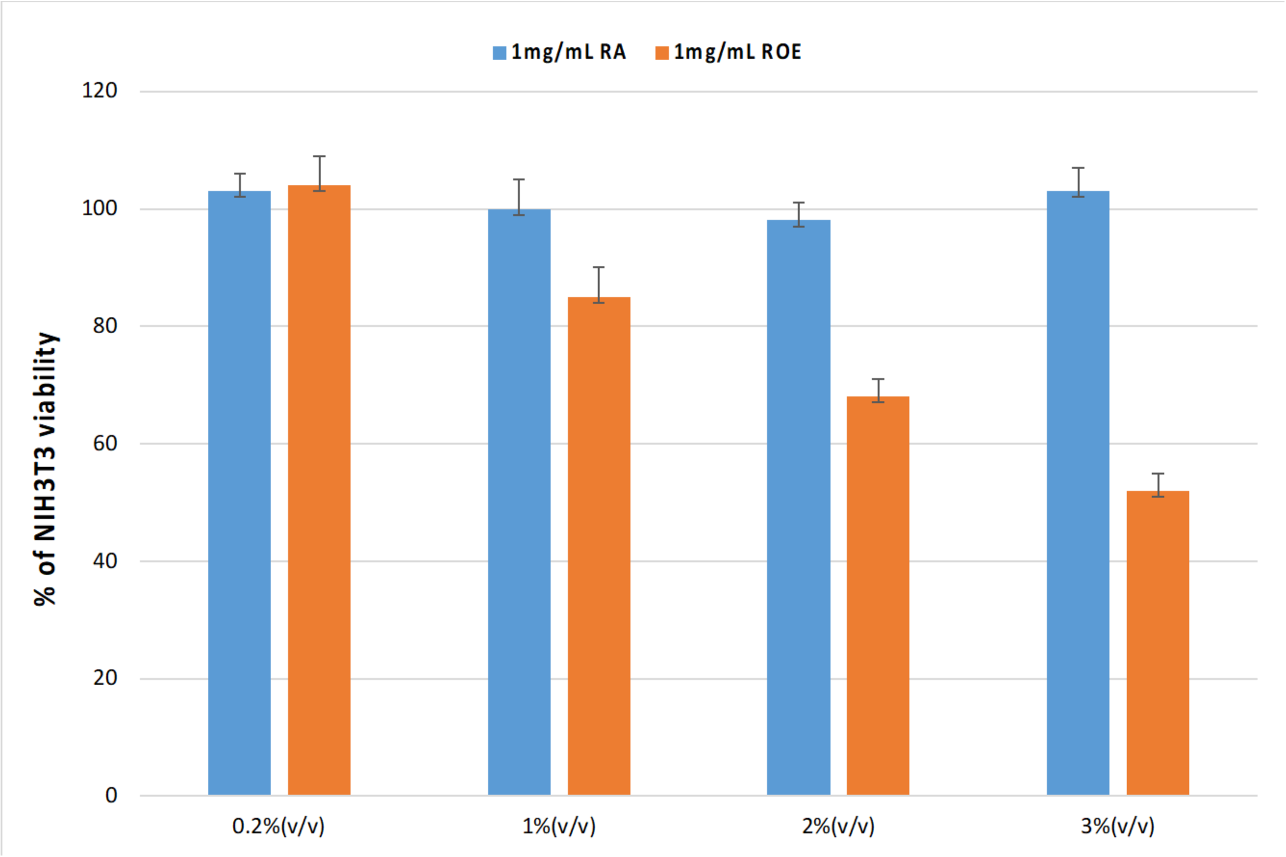

Figure S4. Percentage of viable NIH3T3 after 24 h of contact with different concentrations of RA and ROE as determined by the neutral red uptake. Data are mean SD of three experiments run in six replicates.

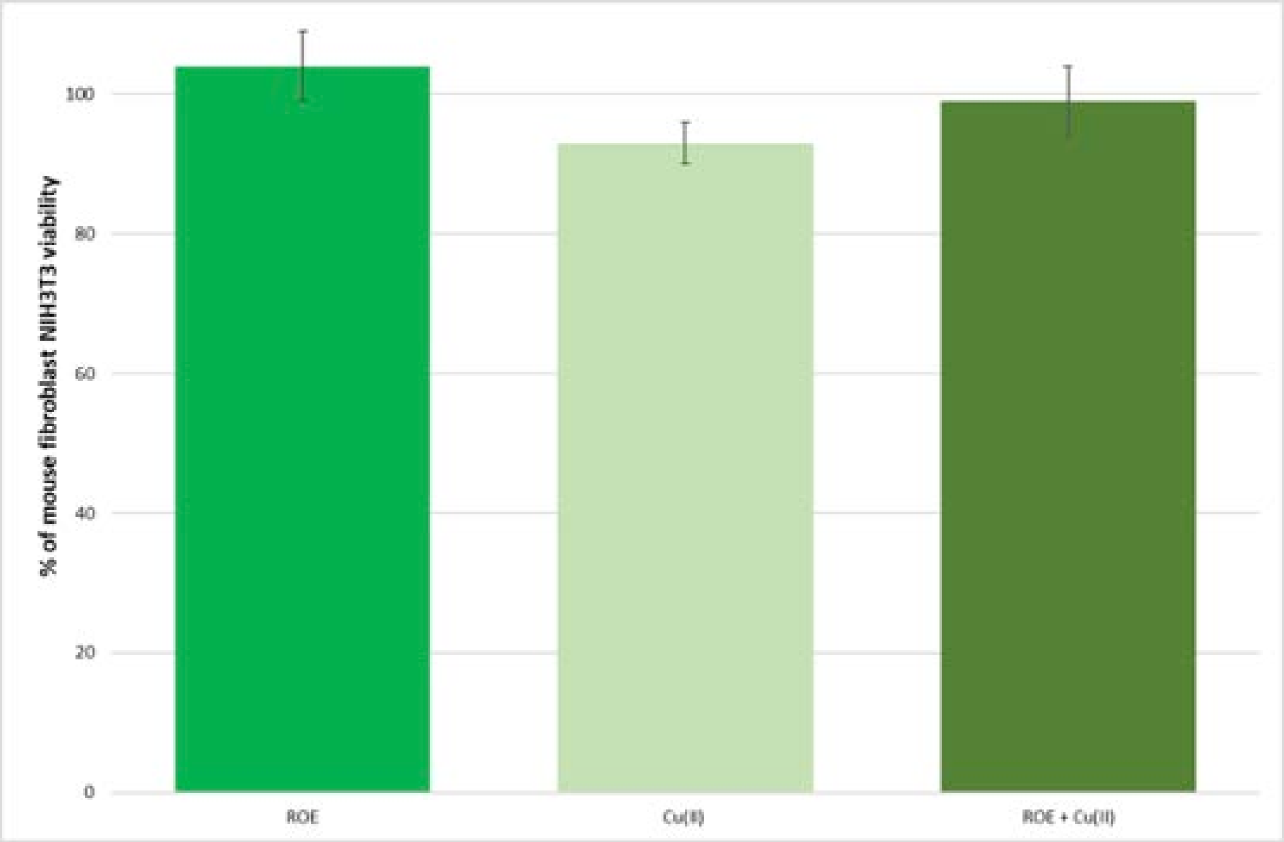

Figure S5. Percentage of viable NIH3T3 after 24 h of contact with ROE 1mg/ml 0.2%(v/v), Cu(II) 5mM 0.2% (v/v) and ROE+Cu(II) as determined by the neutral red uptake. Data are mean SD of three experiments run in six replicates.
